# How epigenetic clocks tick: Unpacking the black box by deciphering biological pathways and transcriptomic signatures of accelerated aging

**DOI:** 10.21203/rs.3.rs-8844558/v1

**Published:** 2026-03-16

**Authors:** Thalida Em Arpawong, Steve Cole, Harshanna Badhesha, Jung Ki Kim, Christopher R. Beam, Eric T. Klopack, Kimberly Siegmund, Bharat Thyagarajan, Eileen M. Crimmins

**Affiliations:** a Leonard Davis School of Gerontology, University of Southern California, Los Angeles, CA, USA; b David Geffen School of Medicine, University of California, Los Angeles, CA, USA; c Dornsife College of Arts and Sciences, University of Southern California, Los Angeles, CA, USA; d School of Public Health, Indiana University, Bloomington, IN, USA; e Division of Biostatistics, Keck School of Medicine, University of Southern California, Los Angeles, CA, USA; f Department of Laboratory Medicine and Pathology, University of Minnesota, Minneapolis, MN, USA

## Abstract

Epigenetic clocks derived from DNA methylation robustly predict biological aging, health, and mortality, yet differ substantially in their predictive profiles. The biological processes underlying these differences remain poorly understood. Using data from 3,227 participants in the U.S. Health and Retirement Study, with contemporaneous DNA methylation and RNA-sequencing, we examined the five most widely used epigenetic clocks (Horvath, Hannum, PhenoAge, GrimAge, and DunedinPACE). Differential gene expression analyses identified clock-specific transcriptional signatures and enriched biological pathways, revealing substantial heterogeneity in the molecular processes captured by each clock. We further derived transcriptomic aging gene scores (TAGS), from differentially expressed genes of each age acceleration clock, and evaluated their associations with aging-related phenotypes. TAGS complemented DNAm clocks, and in several cases, showed stronger associations with age-related morbidities and mortality. Findings revealed more unique than common biological processes underlying clocks, illuminate their internal mechanisms, and advance their interpretability for aging research and clinical applications.

## Introduction

Biological aging occurs at different rates across individuals. Epigenetic modifications—chemical changes to DNA that regulate gene activity--capture measurable DNA methylation (DNAm) patterns across genomic loci and provide useful metrics of biological age^1–5^. These DNAm-based “epigenetic clocks” outperform chronological age in predicting health outcomes, including disease^1–6^ and all-cause mortality^5–8^. Although presumed to reflect aging-related changes in gene regulation^9,10^, these clocks are developed using machine learning approaches applied to genome-wide epigenetic data^11^. Thus, they result in black box constructs leaving the underlying biological mechanisms ambiguous^12^. The specific biological pathways and processes represented by each clock and reasons for their predictive capacity for age-related health and disease remained poorly understood^12,13^.

Epigenetic clocks are increasingly being applied in clinical and commercial contexts to estimate an individual’s rate of biological aging. Although the clocks correlate with health outcomes, the associations vary across physiological^3–5,12,14^, cognitive^15,16^, and mortality outcomes^5–8,12,14,17–19^, even after accounting for genetic^20^ and behavioral factors^21^. These differences likely reflect each clock’s distinct training targets: first-generation clocks were optimized to predict chronological age, second-generation clocks to predict age-related disease and dysfunction, including time-to-death, and third-generation clocks to capture longitudinal change in health or organ system functioning. Among the first-generation clocks, Horvath associates strongly with frailty whereas Hannum is more closely linked to immune responses^22,23^. Studies have found Hannum linked to physical and cognitive functioning whereas Horvath was not^24^, although both associate with metabolic conditions, BMI^25^, and mortality^21^. Second-generation PhenoAge better predicts all-cause mortality, cardiovascular disease, multi-morbidity, and cognitive functioning^4^ while GrimAge predicts frailty, gait speed, and cognitive functioning, and is consistently the strongest epigenetic predictor of mortality^6,21^. Third-generation DunedinPACE predicts the pace of physiological and functional decline across multiple systems^26^. Variability in predictive performance likely reflects differences in epigenetic sites selected by each machine-learning model and specific biological pathways of aging they represent^27,28^.

Epigenetic modifications affect health and aging by regulating gene expression and downstream protein profiles that mediate cellular function. Methylation sites encompassed by epigenetic clocks are typically situated in gene promoters, enhancers, and polycomb-controlled regions^3,29^—key areas of transcriptional control—yet, methylation at specific loci has not shown strong or consistent correlations with expression of nearby genes^30–33^. Studies linking DNAm clocks and gene expression have focused on specific tissues^3^, in vitro models^34^, or comparing exposure groups (e.g., smokers and non-smokers) in single tissue^35^. Such work has implicated that Horvath clock sites map to processes of developmental biology essential to organismal growth and maintenance^12,36^, PhenoAge sites reflect cellular senescence-related pathways^34^, and GrimAge reflects genes enriched in pathways involving morphology and cellular/tissue structure^35^. A deeper understanding is needed on how methylation levels in the most widely used epigenetic clocks correspond to transcriptional regulation and biological processes underlying aging.

The outcome we use in the current study is *epigenetic age acceleration*, which indexes whether individuals are aging more rapidly or more slowly than would be predicted by their chronological age. It is derived by taking the residuals from regressing the clock on chronological age, with positive values indicating faster and negative values slower biological aging. This metric has become central to geroscience efforts to develop biomarkers that capture aging-related decline, and predict health and mortality^37^. Additionally, the recent development of transcriptomic clocks has further advanced the field. These are analogous to epigenetic clocks, but based on gene expression. While epigenetic clocks quantify stable regulatory changes in DNA methylation, transcriptomic clocks capture the functional output of genomic regulation and have emerged as promising complements to epigenetic clocks that improve prediction of multiple aging-related health outcomes^38,39^.

In this study, we investigated how epigenetic age acceleration clocks capture transcriptional signatures of aging and relate to health outcomes in later life. Using data from a population-based sample of older adults in the U.S. Health and Retirement Study (HRS) Venous Blood Study, we analyzed contemporaneous DNAm and gene expression profiles to link molecular aging measures with gene regulatory activity. We first identified genes for which expression levels were associated with each epigenetic age acceleration clock and characterized the biological pathways and processes these genes represent. We then compared clocks to determine whether they reflect shared or distinct aging-related pathways and processes, providing insight into their divergent associations with health outcomes. Finally, we assessed how well transcriptomic signatures, as functional readouts of genomic regulation, reflect the more stable epigenetic regulatory marks. We evaluated whether transcriptomic profiles linked to each clock recapitulate clock-health associations, including with mortality, by constructing additive transcriptomic aging gene scores and testing their predictive value for aging-related phenotypes.

## Results

### Study overview

**Figure 1.**
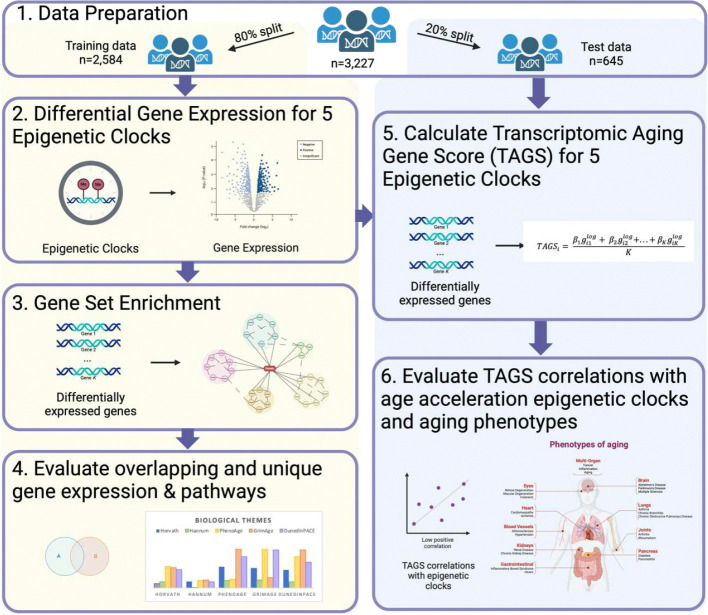
Study workflow. The workflow starts with Step 1: data preparation to randomly separate the full sample into an 80% training (yellow shaded) and 20% test set (blue shaded) for the sequence of steps conducted within each subsample; Step 2: conduct differential gene expression scans on epigenetic age acceleration clocks in the training data; Step 3: evaluate gene set enrichment for both gene ontologies and biological pathways from Step 2 results; Step 4: evaluate overlapping and unique gene biological pathways and processes; Step 5: calculate transcriptomic aging gene scores in the test data from differentially expressed genes identified in Step 2; and Step 6: evaluate the degree that differentially expressed genes, in aggregate, reflect the epigenetic age acceleration clocks and correlate with aging outcomes in the test sample. Created with BioRender.com.

We first calculated epigenetic age acceleration measures in the n=4,018 participants with DNA methylation (DNAm) data and restricted analyses to those with transcriptomics and covariate data, n=3,227. The study workflow is illustrated in Figure 1, with sample characteristics provided in Table 1. In the data preparation step 1, the analytic sample was randomly split into an 80% training set (n=2,584) and 20% hold-out test set (n=645). Both training and test samples included multiple race/ethnicities, 58% women, and mean chronological age of 70. In subsequent steps of the workflow, we identified genes whose expression levels were correlated with each epigenetic age acceleration clock via differential gene expression (DGE) analysis using the training data. Gene transcripts were used as predictors and each epigenetic age acceleration clock as an outcome (step 2). We then used DGE results for each clock for gene set enrichment analyses (GSEA) to determine which biological pathways and processes are enriched for each clock (step 3). Next, we quantified overlap and divergence in enriched biological pathways and processes across clocks (step 4) and finally, using the test data, we constructed transcriptomic aging gene score (TAGS) from clock-associated genes (step 5), and evaluated their correlations with epigenetic age acceleration clocks and health phenotypes (step 6).

#### Differentially expressed genes (DEGs)

**Figure 2.**
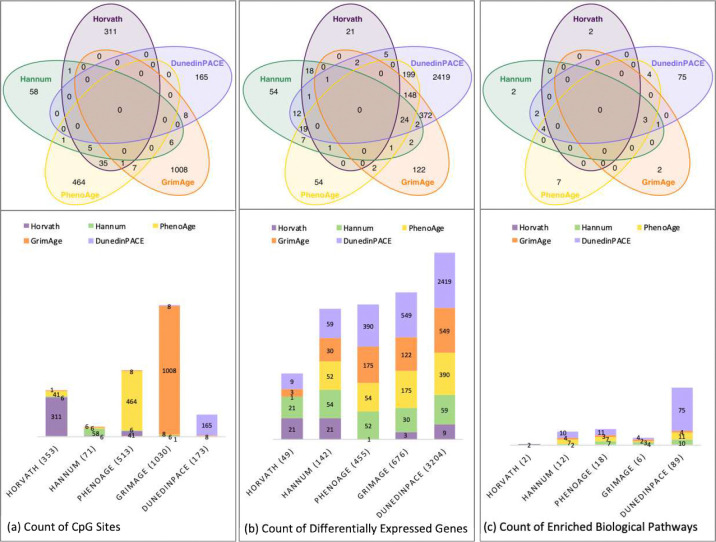
Common and unique features for each DNAm clock. Features are depicted by Venn diagrams and bar plots for (a) the overlapping and unique methylated sites (CpGs) for Horvath (violet), Hannum (green), PhenoAge (gold), GrimAge (orange), and DunedinPACE (purple); (b) the significant differentially expressed genes (DEGs, at FDR p<.01); and (c) the count of enriched biological pathways implicated from GSEA when using the Reactome Knowledgebase. Venn diagrams show that no CpG sites overlapped across all clocks; each clock consisted largely of unique sites, with 1 to 35 sites in common between any of the clocks. No differentially expressed genes were in common across all epigenetic age acceleration clocks, but 816 of 3486 total DEGs were in common between two or more of the clocks. Zero overlapping biological pathways were enriched across all clocks. Up to 14 pathways were shared between at least two clocks. Vertical bar plots show the number of overlapping features for each clock with each of the other clocks. The bar height represents the total count of features (with DEGs in 2b shown on a log-scale to better enable visualization due to large differences in smaller and larger counts) and the x-axis indicates the DNAm age acceleration clock with the total number of associated features in parenthesis; color within the bars represents the degree of overlap of each feature with the other clocks. Clocks that have overlapping features with itself indicates the feature is unique to that clock.

The number of differentially expressed genes (FDR p<0.01) differed substantially by epigenetic age acceleration clock (Figure 1a; SI Tables S1a-S1e) ranging from 49 for Horvath, 142 for Hannum, 455 for PhenoAge, 676 for GrimAge, to 3,204 for DunedinPACE; this contrasts to the 353, 71, 513, 1,030, and 173 epigenetic sites, or cytosine–guanine dinucleotides (CpGs) comprising each clock, respectively, indicating that transcriptomic associations were not proportional to clock size. Overlap in DEGs across clocks was limited with no DEGs shared by all clocks (Figure 2b).

Each age acceleration clock varied in the number of unique DEGs, with 21 of 49 (43%) for Horvath, 54 of 142 (38%) for Hannum, 54 of 455 (12%) for PhenoAge, 122 of 676 (18%) for GrimAge, and 2419 of 3204 (75%) unique to DunedinPACE. The most substantial overlap in DEGs was observed between 2^nd^ generation clocks GrimAge and DunedinPACE with 549 DEGs, PhenoAge and DunedinPACE with 390 DEGs, and GrimAge and PhenoAge with 175 DEGs in common (Fig. 2b). Notably, 86% and 81% of the DEGs for PhenoAge and GrimAge overlapped with DunedinPACE, whereas fewer than 18% of DunedinPACE DEGs overlapped with any other clock. About 43% of Horvath DEGs overlapped with Hannum (both 1^st^ generation clocks), 37 and 42% of Hannum DEGs overlapped with PhenoAge and DunedinPACE, and 38% of PhenoAge DEGs overlapped with GrimAge (both 2^nd^ generation clocks). Overall, 25 DEGs were shared by 4 clocks, 174 by 3 clocks, and 617 by 2 clocks (SI Table S1f). Given that genes act within coordinated networks (i.e., as a pathway), we next examined the biological relevance of clock-associated DEGs using gene set enrichment analyses.

#### Gene set enrichment analysis (GSEA) and biological pathways across clocks

To identify the biological pathways implicated by each clock, we submitted DEGs (FDR<.01; SI Tables S1a-S1e) to Reactome GSEA^40^ (Benjamini-Hochberg FDR<0.25). As shown in Figure 2c, clocks differed dramatically in the number of enriched pathways, ranging from 2 for Horvath to 89 for DunedinPACE (SI Tables S2a-e). Remarkably, despite comparable predictive performance for aging phenotypes, GrimAge and DunedinPACE differed substantially in the number of pathway correlates (6 vs 89). Second-generation PhenoAge and DunedinPACE clocks map to a broader array of RNA pathways than first-generation clocks, potentially explaining their greater predictive power for health outcomes. In contrast, second-generation GrimAge assessed fewer RNA pathways and they predominantly mapped to immune system functions. Differences in the number of detected pathways reflect both biological and statistical factors, including the number of DEGs per clock (i.e., impacting the detectable FDR significance in GSEA).

**Figure 3.**
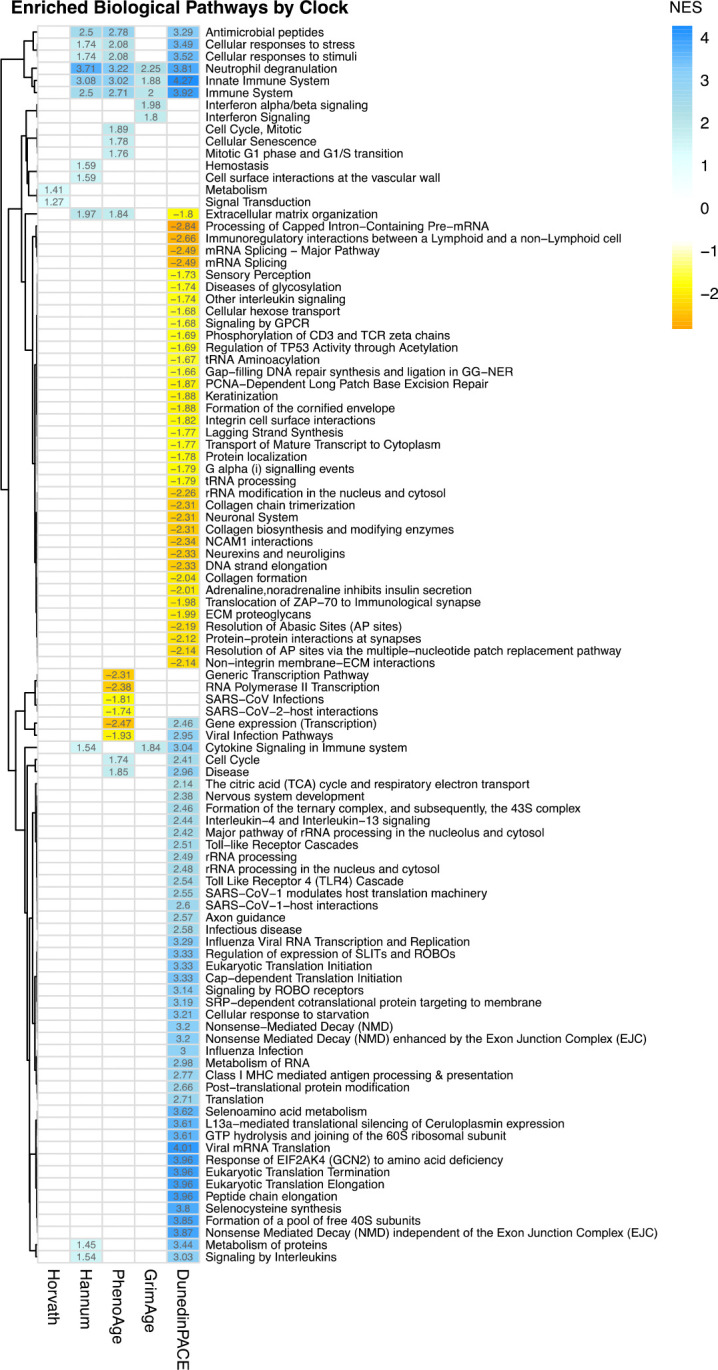
Heatmap of enriched biological pathways in common and unique to each of the five clocks. The normalized enrichment score (NES) for the pathway is shown. A positive NES indicates increased activity of that pathway whereas a negative NES indicates decreased activity. Correlation-based hierarchical clustering of the pathways is shown on the left.

Clocks also differed in pathway specificity (Figure 3). No pathways were common to all clocks (Figure 2c), although 14 were shared by at least two clocks. Three immune-related pathways–neutrophil degranulation, innate immune system, and immune system–were upregulated for Hannum, PhenoAge, GrimAge, and DunedinPACE. Additional overlap included antimicrobial peptides, cellular stress response, cellular response to stimulus, extracellular matrix, and cytokine signaling pathways across subsets of clocks.

Each clock also showed distinct signatures: Horvath was enriched for metabolism and signal transduction; Hannum for homeostasis and vascular wall interactions; GrimAge for Interferon signaling; PhenoAge for activated cellular senescence and mitotic pathways alongside repression of transcriptional and viral interaction pathways. DunedinPACE exhibited the most extensive profile, with activation of pathways spanning protein metabolism, immune signaling, nervous system development, and cellular respiration, and repression of pathways related to DNA repair, G protein-coupled receptor signaling, ECM organization, and neuronal processes. Gene expression and viral infection pathways were downregulated for PhenoAge, but upregulated for DunedinPACE whereas ECM organization exhibited the opposite pattern; it was upregulated for PhenoAge and Hannum but not DunedinPACE.

#### Gene Ontologies (GO) and biological processes across epigenetic clocks

**Figure 4.**
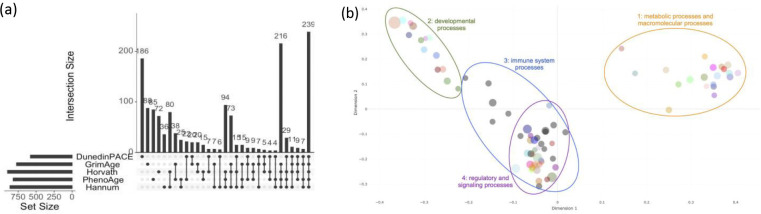
Comparisons of Gene Ontologies (GO) across clocks identifies common functional clusters across clocks. (a) The upset plot shows the number of overlapping and unique enriched GO terms between age acceleration clocks, from the total of 1,453 GO terms identified (SI Table S3). (b) A multi-dimensional scaling (MDS) plot is shown, constructed based on semantic similarity distance representing proximities of the 75 functional clusters of biological processes, derived from aggregating the 1,453 enriched GO terms using hierarchical clustering. Dots represent clusters with size proportional to the number of GO terms in the cluster. The MDS plot reveals four main functional profiles based on cluster similarity and aggregation.

To evaluate broader functional activities associated with clock-linked transcriptional changes, we conducted GSEA of GO terms biological processes (Ensembl, release 112) using ViSEAGO enabling direct comparison across clocks. Across all clocks, 1,453 GO terms (SI Table S3) were enriched for at least one clock, of which 467 (32%) were unique to a single clock (Figure 4a). In contrast to the large differences observed for Reactome pathways, the number of enriched GO terms was relatively similar across clocks (898 Horvath, 864 Hannum, 820 PhenoAge, 773 GrimAge, and 580 DunedinPACE) indicating greater convergence at the level of general biological processes. In terms of overlap, there were 239 (16%) GO terms enriched across all 5 clocks, and 272 in common across at least 4 clocks, with 216 of those being in common to Horvath, Hannum, GrimAge and PhenoAge (Figure 4a). Whereas Reactome analyses highlighted marked differences in the number and identity of clock-specific pathway signatures, GO enrichment revealed considerably greater overlap across clocks, indicating convergence at the level of higher-order biological processes.

To synthesize the functional meaning of the enriched GO terms, we reduced redundancy using semantic similarity-based hierarchical clustering (detailed steps in SI), yielding 75 GO clusters (SI Fig S2). Multi-dimensional scaling (MDS) of cluster proximities revealed four major functional theme sets common across clocks (Figure 4b): (1) Metabolic processes and macromolecular processes encompassing DNA, nucleic acid, and protein metabolism (20 clusters from 341 GO terms); (2) Developmental processes, including blood cell development, neurogenesis, and organ and tissue development (14 clusters from 289 GO terms); (3) Immune system processes, such as leukocyte activation, lymphocyte differentiation, and regulation of cell motility (16 clusters from 287 GO terms); and (4) Regulatory and signaling processes, including cytokine and interferon signaling, regulation of immune and inflammatory responses, apoptotic signaling, transport, and signal transduction (25 clusters from 536 GO terms).

#### Transcriptomic Aging Gene Scores (TAGS) and Associations with Aging-Related Phenotypes

**Figure 5.**
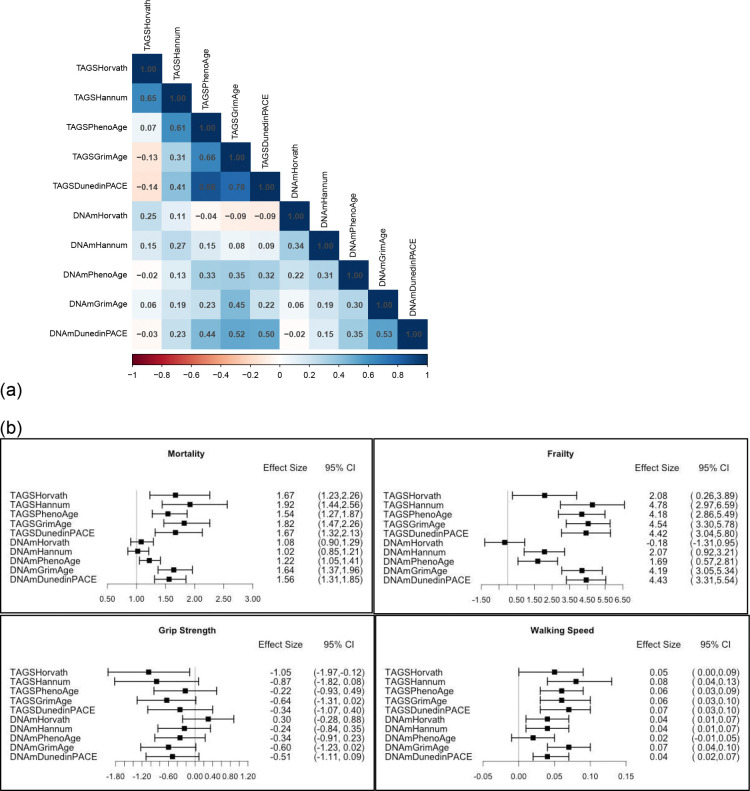

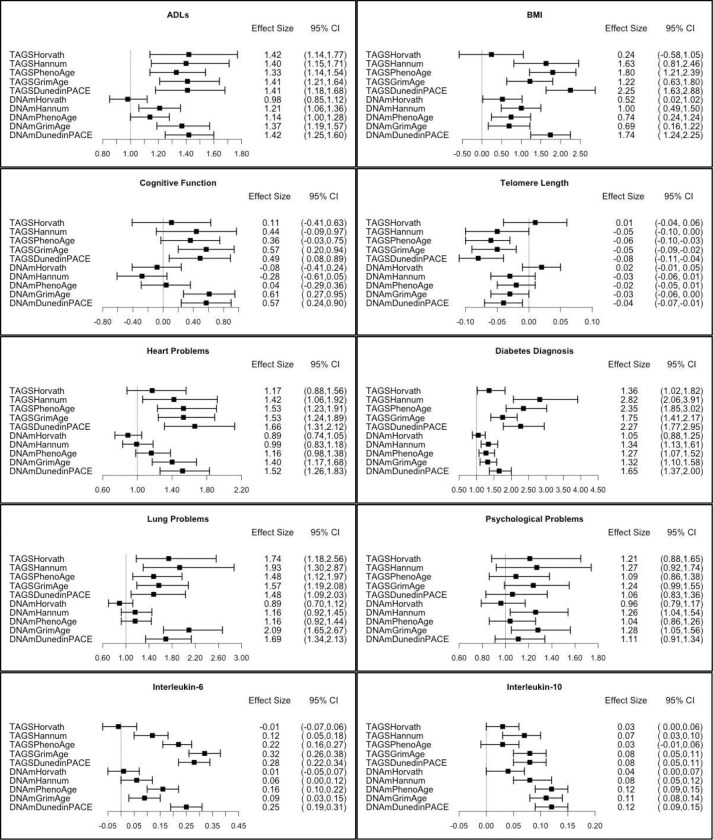
Relationships between epigenetic clocks and transcriptomic aging signatures with health outcomes. Relationships are shown between (a) gene expression signatures, called transcriptomic aging gene scores (TAGS), and epigenetic clocks (with prefix DNAm), and (b) TAGS and epigenetic age acceleration clocks, and aging-related outcomes. Each row represents the effect size from a separate statistical model. Each epigenetic clock and TAGS score included in the models was residualized for age and cell type. Effect sizes represent a Hazard Ratio for mortality and unstandardized beta coefficients for grip strength, walking speed, BMI, cognitive function, telomere length ratio, interleukin-6 and interleukin-10; and rate ratios for levels of frailty, having activities of daily living (ADLS) difficulties, and diagnoses of heart problems, diabetes, lung or psychological problems. Prediction models were evaluated in hold-out validation sample, adjusted for covariates (sex, smoking, ancestry principal components, batch, and additionally height for grip strength and walking speed).

To address whether active gene regulatory levels, via gene expression, recapitulates regulatory changes indicated by epigenetic clocks and associations with health phenotypes, we construct a transcriptomic aging gene score (TAGS) for each epigenetic age acceleration clock and test its predictive power in the hold-out test sample, n=645 (sample characteristics in SI Appendix Table S4). Each DNAm age acceleration clock correlated with its TAGS at r=.25 for Horvath, r=.27 for Hannum, r=.33 for PhenoAge, r=.45 for GrimAge, and r=.50 for DunedinPACE (Figure 5a). The varying correlations between individual TAGSs and specific age acceleration clocks likely stem in large part from the varying number of DEG correlates of each clock, and thus the level of statistical power/score reliability that can be achieved as a result. TAGSs correlated with each other at r=−0.14 to .86 whereas DNAm age acceleration clocks correlated with each other at r=−0.02 to 0.53. Phenotypes tested for association with TAGS were based on aging-related functional ability and additional metrics informed by enriched biological pathways found (e.g., metabolism, immune system). As shown in Figure 5b, generally, TAGS showed larger effects in predicting health and aging outcomes compared to epigenetic age acceleration clocks. This was true for ADLs, walking speed, frailty, heart problems, mortality, lung problems, diabetes diagnoses, telomere length, and a pro-inflammatory cytokine interleukin-6 (IL-6). TAGS were not significant predictors for psychological problems, and were mixed with respect to grip strength, cognitive functioning and anti-inflammatory cytokine interleukin-10 (IL-10).

**Figure 6.**
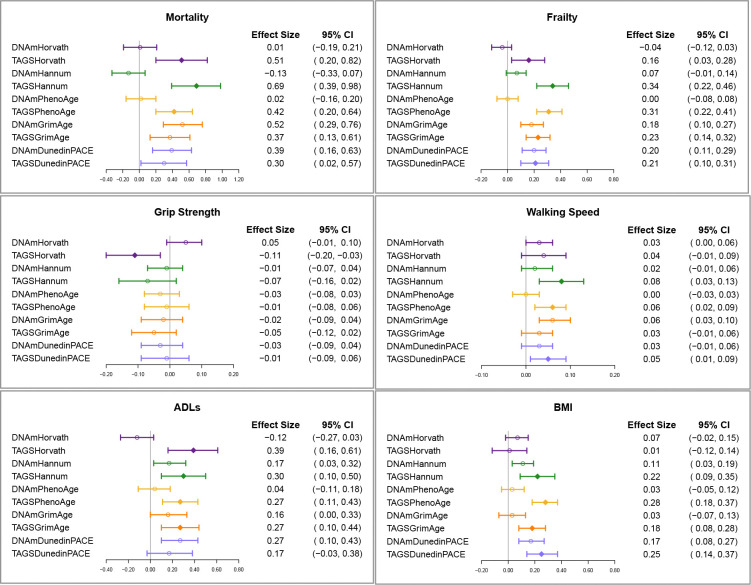

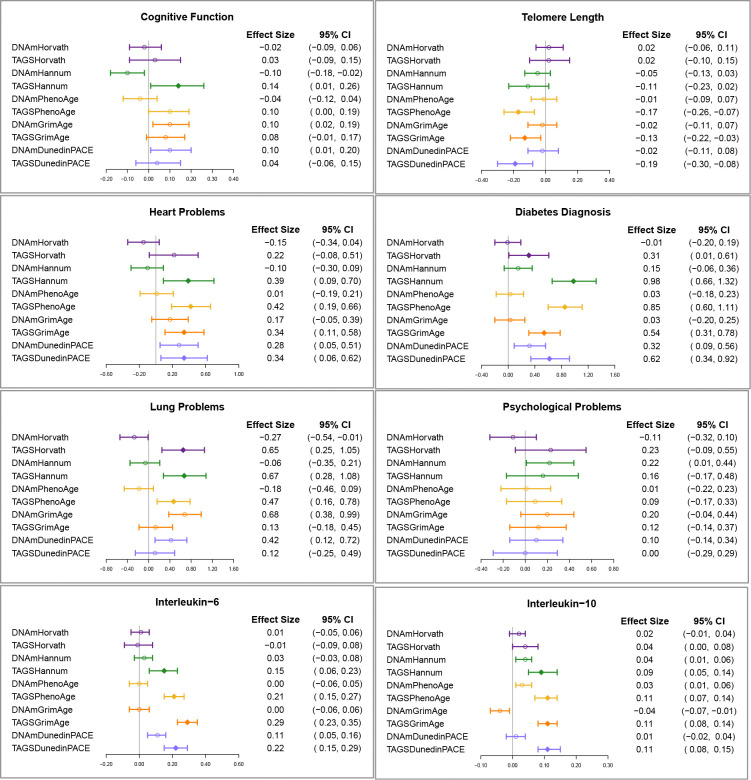
Relationships between epigenetic and transcriptomic clocks with aging-related health outcomes. Relationships are shown in models that include both TAGS and epigenetic age acceleration clocks (each residualized for age and cell type), with separate models run for each outcome. Five statistical models were run for each outcome with color groups indicating clock predictors run in the same model. Plots show standardized betas to compare RNA and DNAm clock prediction with open circles marking the effect sizes, except where the TAGS effect size was both significant and stronger than the DNAm effect size and a filled diamond is used. Prediction models were evaluated in the hold-out validation sample, adjusted for covariates (sex, smoking, ancestry principal components, batch, and additionally height for grip strength and walking speed).

Next, we tested predictive value of TAGS with aging-related health outcomes with the respective DNAm age acceleration clock in the same model (Figure 6). For mortality, frailty, ADLs, and IL-10, the TAGS out-performs DNAm for each of the first-generation clocks, but for the second-generation clocks, both the TAGS and DNAm provide some significant distinctive contributions (i.e., TAGS and DNAm are complementary predictors). For mortality, the distinctive contribution from DNAm is generally greater than that of TAGS whereas for frailty, ADLs, and BMI, the opposite is true with the contribution from TAGS being generally greater. For diabetes, heart problems, and IL-6, generally TAGS remained significant whereas DNAm age acceleration clocks did not when in the same model, except for DunedinPACE in which both clock versions made distinctive contributions.

## Discussion

The gene regulatory dynamics assessed by epigenetic clocks remain poorly understood, creating ambiguity on what these clocks represent at the level of biology and geroscience. In this study, we show that epigenetic age acceleration clocks differ markedly in their associated gene expression correlates and implicated biological processes, highlighting the distinct dimensions each clock captures to reflect accelerated epigenetic aging. These differences are both quantitative (in the dramatically differing number of DEGs associated with different clocks) and qualitative (in the relatively sparse overlap across clocks in their specific DEG correlates as well as the specific Reactome biological pathways they represent). Despite this heterogeneity, gene set enrichment analysis (GSEA) also revealed convergence on key aging-related biological processes (Gene Ontology), suggesting both diversity and complementarity among clocks. These findings offer insight into clock-specific relevance in geroscience research. For example, while Hannum and later generation clocks associate broadly with immune, metabolic, and functional aging phenotypes, each demonstrates distinct predictive strengths for specific health outcomes. In this analysis, we find that they each demonstrate distinctive biological correlates as well, and this may account for their differing patterns of predictive strength. By leveraging DNA methylation and RNAseq data collected contemporaneously from a cohort of older adults, our study clarifies both the shared and unique biological signatures underlying accelerated epigenetic aging.

### How are epigenetic clocks associated with gene expression levels?

The relationship between DNA methylation and gene expression in aging remains complex, yet studying their co-variation—particularly in blood-derived data from older adults—offers insight into the systemic regulatory processes associated with biological aging. As discussed by Horvath and Raj (2018), epigenetic clocks draw on distinct sets of CpG sites, many of which may be functionally unconnected^12^. This is consistent with our finding that 82–98% of CpG sites used in each clock are unique, reflecting the differing design principles and training targets across clocks. Field et al. (2018) emphasized that clocks vary not only in their methodological construction (e.g., training targets and CpG selection criteria) but also in their biological relevance—particularly whether they reflect causal mechanisms or merely correlate with aging phenotypes^29^. Given this divergence, it is not surprising that the transcriptomic profiles associated with each age acceleration clock also vary substantially. Our analysis revealed that the number of differentially expressed genes (DEGs) ranged from as few as 49 for Horvath’s first-generation clock to over 3,200 for DunedinPACE, a third-generation clock designed to predict the pace of aging. Consistent with Field et al.’s categorization, second- and third-generation clocks—which are trained to predict health and mortality-related phenotypes rather than just chronological age—showed greater transcriptomic concordance; for instance, GrimAge and DunedinPACE shared 549 DEGs (approximately 81% of total DEGs in GrimAge and 17% of DunedinPACE). In contrast, first-generation clocks displayed limited overlap both in CpG composition and downstream gene expression, supporting the view that they capture more general temporal features of aging rather than mechanistic or health-related aging processes. Additionally, canonical features of aging may be underrepresented in array-based CpG selection during clock development, as key regulatory sites marking global aging processes may reside outside promoters and gene bodies, which dominate EPIC array coverage. Together, these findings underscore the heterogeneity in the biological signals captured by different epigenetic age acceleration clocks.

### To what extent do clocks tap overlapping vs. distinct biological pathways and processes?

Despite the gene-level heterogeneity and modest overlap, GSEA analyses of higher order gene sets (i.e., Reactome pathways and Gene Ontology biological processes) identified convergence on certain biological pathways, particularly around immune and stress-response functions. Immune-related pathways such as neutrophil degranulation and innate immune system activation were commonly upregulated in Hannum, PhenoAge, GrimAge, and DunedinPACE. GO enrichment analysis clarified collective patterns across clocks on the molecular level. Although 32% of GO terms were unique to a single clock, 16% were shared across all five and clustering of 1,453 enriched GO terms revealed four major biological themes common across clocks: metabolic processes, developmental processes, immune system processes, and regulatory and signaling processes. These shared functions suggest that while clocks diverge at the level of specific genes and biological pathways, they tap into some of the same fundamental biological processes of aging. Those that stand out are the immunologic commonalities across clocks, including neutrophil/inflammatory biology, cytokine activity, and particularly interferon activity, suggesting that these are critical correlates of biological aging. These collective biological themes across clocks offer insight into why, despite different CpGs and genes being marked by the clocks, they associate with some of the same aging phenotypes. Beyond the commonalities, each clock exhibited a distinct enrichment profile discussed by clock, below.

The Horvath clock, trained on chronological age across tissues^3^, was associated with few and a limited set of biological pathways, primarily metabolism and signal transduction. These core homeostatic processes provide a mechanistic link to the clock’s established associations with frailty, metabolic dysfunction, and mortality. Disruption of metabolic and signaling networks (e.g., insulin/IGF-1, mTOR, AMPK) may impair stress responses and tissue maintenance, suggesting the Horvath clock captures cumulative epigenetic destabilization of fundamental aging pathways.

The blood-based Hannum clock, also trained on age^2^, showed substantially more DEGs and broader pathway enrichment than Horvath, particularly in immune, inflammatory, and vascular-related processes. Enrichment for innate and adaptive immunity, cytokine signaling, neutrophil degranulation, and vascular wall interactions indicates Hannum reflects primarily immunosenescence and inflammaging (i.e., declining immune surveillance, reduced pathogen defense, and increased chronic inflammation^41,42^). Additional associations with extracellular matrix remodeling and protein metabolism link this clock to declines in tissue integrity, regenerative capacity, and thus decline in physical function. Pathways associated with chronic inflammation, vascular dysfunction (reflected in vascular wall interactions), and impaired cellular homeostasis link the clock to metabolic and cardiovascular health, suggesting the Hannum clock reflects a composite of interconnected physiological systems, accounting for its predictive value across aging-related outcomes.

PhenoAge, trained on clinical biomarkers of mortality and healthspan^4^ was associated with extensive transcriptomic changes implicating immune activation, cellular senescence, mitosis, neutrophil degranulation, innate immunity, and cellular stress response. These activated pathways align with known hallmarks of aging, where cellular senescence drives chronic inflammation (inflammaging), impaired tissue repair, accelerating multi-system decline^43–45^. Concurrent repression of transcriptional and antiviral response pathways further suggests reduced protective capacity with aging. Together, these findings indicate that PhenoAge integrates signatures of both accumulated damage and declining resilience, consistent with its strong associations with multimorbidity and lifespan.

GrimAge was associated with many DEGs (more than PhenoAge), but paradoxically with a substantially fewer Reactome pathways and GO biological functions. This suggests that GrimAge may be more internally heterogeneous than other clocks, with many empirically predictive CpG elements but few coherent overall biological themes. The pathways identified converged on innate immune and inflammatory pathways, including interferon signaling, neutrophil degranulation, and cytokine signaling–all supporting its association with inflammaging^46,47^. Chronic activation of these pathways likely underlies GrimAge’s strong prediction of frailty, cognitive decline, and mortality, reflecting the central role of pro-inflammatory and immune dysregulation in aging, despite the fact that it was not trained on any biomarker of this specific process.

DunedinPACE showed the most extensive transcriptomic and pathway associations, consistent with its design to capture the rate of multi-system physiological decline^26^. Activation of pro-aging pathways reflects heightened cellular burden and inflammation, including transcriptional and protein metabolic processes, mitochondrial activity (cellular respiration), immune signaling, and cellular stress response. The dual signature of repression of pro-health pathways (core homeostatic functions of repair and regulation) provides a mechanistic basis for DunedinPACE reflecting biological decline and underlies its association with multiple functional impairments. DunedinPACE also showed the opposite association with ECM organization compared with PhenoAge and Hannum, likely reflecting a biological shift away from structural maintenance toward immune and stress-response processes.

### Summary across clocks pertaining to potential clinical research use

Given the distinct biological pathways and aging phenotypes associated with each epigenetic age acceleration clock, different clocks may be better suited for evaluating specific aging-related and health interventions. Horvath’s links to metabolic and signal transduction pathways suggesting particular relevance to interventions targeting metabolic health, such as caloric restriction, dietary modification, and pharmacologic regulation, while its associations with frailty, BMI, and mortality support its use for broad aging-risk assessment. Hannum’s clock, more closely tied to immune and inflammatory pathways, including neutrophil degranulation and cytokine signaling, makes it particularly suited for studies targeting immune senescence, chronic inflammation, or vascular aging, such as anti-inflammatory or immunomodulatory therapies. PhenoAge, which integrates signals from cellular senescence, cell cycle regulation, and systemic inflammatory response, aligns with interventions aimed at reducing multi-morbidity risk, or supporting biological resilience. GrimAge, reflecting interferon signaling and immune surveillance, is the strongest predictor of mortality and functional decline, making it valuable for prognostic monitoring and late-life interventions or inflammation-targeting therapies. Finally, DunedinPACE reflects a broad set of aging-related processes, including immune activity, mitochondrial function, protein metabolism, and neural pathways, and appears sensitive to change in functional outcomes like gait speed, ADLs, balance, and cognitive functioning, making it useful for tracking the impact of exercise and geroprotectors to slow the pace of aging. Overall, clock suitability in clinical research depends on the biological pathways targeted by the intervention and functional outcomes of interest.

### How well do the downstream gene expression levels associated with each clock recapitulate the associations with health phenotypes of aging, including mortality?

Because RNA reflects active gene expression, transcriptomic profiles provide a dynamic readout of biological aging^48^. Consistent with this, Transcriptomic Aging Gene Scores (TAGS) showed moderate correspondence with their parent epigenetic clocks, yet in several cases more strongly predicted functional, clinical, and mortality outcomes. The improved performance of TAGS for phenotypes related to metabolic health, frailty, cardiovascular health, immune function and survival suggests that clock-associated transcriptional activity captures aging-related processes not fully reflected by DNA methylation alone. At the same time, the complementary predictive value of TAGS and epigenetic clocks for several outcomes indicates that DNAm clocks encode meaningful regulatory information, likely acting in trans (vs cis) to shape downstream gene expression. Together these findings support transcriptomic clocks such as TAGS as a valuable extension of epigenetic aging measures and could be routinely used as an enhancement of the DNAm predictor for future research aimed at achieving either optimal prediction of aging phenotypes or a better understanding of biological mechanisms underlying aging.

Given that epigenetic clocks have emerged as markers of both healthspan and lifespan, serving as indicators of one’s ticking time, their association with mortality holds particular significance in geroscience. Notably, while all five *transcriptomic* clocks predicted all-cause mortality, the age acceleration *epigenetic* versions for second-generation clocks also did, whereas the first-generation age acceleration epigenetic clocks did not. This remarkable discrepancy supports the notion that the epigenetic clocks trained on chronological age may not capture the interindividual variability in the molecular mechanisms driving *accelerated* biological aging and mortality risk. These findings underscore the need to consider the distinct biological dimensions captured by epigenetic versus a growing number of transcriptomic aging clocks, and to further investigate the distinct aspects of aging biology that epigenetic and transcriptomic clocks respectively reflect. Furthermore, incorporating transcriptomic measures thus advances aging research by elucidating the active molecular pathways underlying methylation-based age acceleration and providing a more responsive indicator of biological aging and its modulation through interventions.

### Limitations

This study has limitations, which include that inherent in calculation of epigenetic clocks is the ambiguity on sites being hyper- or hypo-methylated and thus resulting in upregulation or downregulation of associated gene expression. This limits our ability to evaluate whether the transcriptomic scores faithfully reflect the activation or repression of a specific gene’s activity. Additionally, epigenetic patterning in blood may differ from tissue-specific patterning and blood-based interpretations may capture less specificity in biological functionality than in tissues. However, evaluation in blood-based DNA methylation data enabled us to make comparisons using a multi-clock and multi-omic approach. Cell type variations may contribute to biological pathways detected. Thus, we adjusted for cell types to better compare components of the clocks that represent accelerated aging, but could not fully distinguish biological pathways and processes completely unconfounded with cell type. Finally, this study’s TAGS test sample was drawn from the same overall data set as used to develop the TAGS scores in the training data. As such, this study may over-estimate predictive performance that would occur in a test sample from a separate study. Future research using other samples will be important to assess the performance of TAGS. It may be that the TAGS developed here can be readily applied to other samples (e.g., with RNA abundance measured by other methods such as alternative RNAseq protocols or microarray assays). Alternatively, the approach we have taken here to develop TAGS may potentially be applied to other data sets (i.e., developing new sample-specific TAGS within each data set to accommodate its specific RNA and DNAm measurement platforms). In either case, the development of TAGS provides an important new complement to DNAm-based assessments of epigenetic age, and the combination of functional genomic (transcriptomic) and epigenomic (DNAm) assessments of biological aging may well be more predictive and scientifically illuminating than the results of either one in isolation from the other.

### Conclusions

Epigenetic clocks represent a promising avenue for assessing biological aging and its related health outcomes. As the field rapidly progresses, it is crucial to deepen our understanding of the biological mechanisms behind these clocks to pave the way for potential translation into clinical applications. By linking DNA methylation patterns to gene expression and physiological processes, researchers can better grasp the complex biological processes driving accelerated aging. This is a crucial step for deepening our understanding of the mechanisms behind these clocks, thus paving the way for potential translation into clinical applications and developing targeted interventions to slow its pace.

## Methods

### Sample.

Data come from the Health and Retirement Study (HRS), a nationally representative longitudinal study that includes the U.S. population over age 50 years and their spouses. The study began in 1992 and is on-going with biennial waves of data collection^49^. Gene expression levels (n=3,738) and epigenetic clocks (n=4,018) have been derived from the 2016 venous blood subsample from consenting participants. Additionally, genetic ancestry principal components were calculated from genotyping data from 2006–2012. For this study, we use the sample with gene expression, DNA methylation and genetic data, and covariates to yield an analytical sample of n=3,227. As shown in Figure 1, we randomly split the data into an 80% training (n=2,582) and 20% test (n=645) set in order to reserve a sub-sample for validation testing of the transcriptomic scores.

### Gene expression.

In the HRS cohort, RNA samples were sequenced as 50 base pair single read sequences with a minimum of 20 million reads per sample on a NovaSeq with ribosomal and globin messenger RNA reduction completed prior to sequencing. Sequencing was based on the TopMed/GTEX analysis pipeline, first using the STAR aligner^50^ to align RNAseq reads to the GrCh38 reference genome from GENCODE, then calculating quality control metrics using RNASeQC^51^. SAMTools^52^ and RSEM^53^ were used to obtain gene read counts. Gene expression values used were calculated as the log_2_ counts per million (log2cpm). We included 49,072 gene transcripts in the differential gene expression scans.

### DNA methylation.

DNA methylation was assessed using the Infinium Methylation EPIC BeadChip (Illumina, Inc., San Diego, CA). Preprocessing and quality control were completed using the *minfi* package in R, with removal of suboptimal samples or sex mismatched samples thereby yielding 836,660 methylation features^54^. In this study, we used epigenetic clocks calculated by HRS^55^: Horvath^3^, Hannum^2^, PhenoAge^4^, GrimAge^5^, and Dunedin Pace of Aging (DunedinPACE)^1^. Epigenetic clocks were residualized for chronological age, and therefore, positive residual scores indicate accelerated biological aging, whereas negative scores indicate slower aging. Epigenetic clocks were further residualized for cell types (B lymphocytes, CD4^+^ and CD8^+ T^ lymphocytes, granulocytes, monocytes, natural killer cells) to control for cell type variation in peripheral blood^56,57^.

### Covariates for differential gene expression.

Sex at birth was self-reported. Ancestry principal components were used to minimize variation due to genetic ancestry. Additional covariates for TAGS regression included lifetime smoking status, which was self-reported (yes/no) and height in centimeters.

### Health Outcomes

*Mortality* was assessed by death occurring between the time of blood sample collection and the 2022 follow-up survey, which included 123 of 645 participants in the test sample (19.1%) which was similar in proportion to the training sample (19.6%). Survival months ranged from 0 to 90 months(mean=62.58, SD=18.33). Self-reported health conditions, including having ever been diagnosed with diabetes, heart problems, lung problems, or psychiatric problems were coded as binary indicators (0=no, 1=yes).

### Physiological functioning.

Physiological and functional abilities were indexed using five measures: (1) Grip strength was assessed using a hand dynamometer, with two trials per hand and averaged across trials; (2) gait speed was measured as the average time to walk a 98.5-inch course, across two trials only for participants aged 65 and older; (3) activities of daily living (ADLs) were assessed as a count of reported difficulties with dressing, walking across a room, bathing, eating, and getting in or out of bed (range 0–5); (4) frailty index was calculated as a composite of 39 health deficits, including ADLs (5 ADLs above plus toileting) and IADLs (making phone calls, managing money, taking medications, shopping for groceries, and preparing a hot meal), memory abilities (immediate and delayed word recall), psychiatric problems, joint replacement, vision and hearing difficulty, Nagi functioning, and chronic diseases (i.e., hypertension, angina, congestive heart failure, diabetes, stroke, lung disease, arthritis, cancer, heart attack). Binary variables were coded as 0/1 for absence/presence of deficits. Continuous and ordinal variables were rescaled 0–1 to indicate severity. The sum of deficits was divided by the total number of measures and multiplied by 100 (range 0–100).

*Cognitive functioning* was measured using the modified Telephone Interview of Cognitive Status (TICSm), including immediate and delayed word recall (range 0–10 each), serial sevens (0–5), and backwards counting (0–2), for a total score ranging 0–27. The score was reverse-coded to represent cognitive errors.

*Immune system biomarkers* were used to index immune system capacity. Cytokines Interleukin-6 (IL-6) and Interleukin-10 (IL-10) were measured in serum using the respective Simple Plex immunoassay on the ELLA System^58^.

### Statistical Analysis

The overview of statistical steps is shown in Figure 1. For Step 2, the DGE analysis, we estimated the significance of the differences in expression level for each transcript on the age accelerated epigenetic clock using linear regression models in R, version 4.3.1^59^. Because gene expression levels were log_2_-transformed cpm, regression coefficients are interpreted as the expected change in the age acceleration outcome per two-fold increase in gene expression level, adjusted for covariates. Models were adjusted for technical variation (i.e., RNA sequencing batch run), sex, smoking, and five genetic ancestry principal components. Multiple test correction was completed using the fdrtool package in R to estimate the local False Discovery Rate (FDR) with differentially expressed genes defined at a local FDR p-value≤.01^60^. Volcano plots were constructed to show the statistical significance of the change in outcome per doubling of each gene transcript in the analysis; this was done by plotting the regression coefficients by positive or negative valence (Supplemental Figure S1). Overlap between differentially expressed genes by clock was compared using the R VennDiagram package^61^.

#### Gene set enrichment and pathway analysis.

In Step 3 (Figure 1), gene set enrichment analysis (GSEA)^62^ was performed to delineate functional similarities implicated by the differential gene expression levels identified for each clock in Step 2. GSEA assumes that large differences in individual genes have important effects on biological processes, and that the small and coordinated activities in sets of functionally related genes can direct important effects. Thus, differentially expressed gene lists from Step 2 that were used for GSEA were not filtered for size, and selected using an FDR adjusted p<.01 threshold to balance risk of type 1 and type 2 error. With GSEA, we initially identified biological themes implicated by gene sets using the Reactome knowledgebase and ReactomePA package^40^. As standard for GSEA, a Benjamini-Hochberg FDR cut-off=.25 was initially used to identify statistically significantly enriched biological pathways. However, results yielded hundreds of pathways (Horvath 112, Hannum 36, PhenoAge 207, GrimAge 234, DunedinPACE 258) with redundancies (e.g., “Mitotic G1 phase and G1/S transition”, “Mitotic Metaphase and Anaphase”, “Mitotic Anaphase”, “Mitotic G2-G2/M phases”) which is not unexpected from gene sets generated from differential gene expression analysis^63,64^, and for which higher level parent pathways (e.g., Cell Cycle) could otherwise be identified. To reduce redundancies and facilitate interpretation of pathways for each clock, we used WebGestalt^65^ (minimum overlap of 5 genes per category, 1000 permutations) and a FDR<.25 threshold for enrichment (Figure 3).

#### Gene Ontology (GO) enrichment analysis.

In Step 3, to evaluate the degree of overlap in biological processes implicated by each clock, we first identified enriched Gene Ontologies^66,67^ using the topGO package, implemented in ViSEAGO^68^. Enriched GO terms identified across clocks underwent hierarchical clustering (SI Methods) by semantic similarity, and then Ward D2 aggregation criterion was used to identify functional groups of the 75 clusters. Proximities of the clusters were visualized in a Multi-dimensional Scaling (MDS) plot to enable us to distinguish functional profiles and more easily interpret the biological functions carried out by the clustered groups.

#### Transcriptomic aging gene scores (TAGS) for each epigenetic clock.

For direct comparisons on whether epigenetic clock associations recapitulate with gene expression levels (Figure 1, Step 4), the differentially expressed genes identified the FDR adjusted p-value≤.01 were used to create weighted transcriptome scores. A score for the *i*th individual was constructed as the weighted sum of the individual’s log-transformed and normalized gene expression values (in log2 counts per million) of K identified genes, divided by the K number of genes:

$$TAGS_{i}=\frac{\beta_{1}g_{i1}^{log}+\beta_{2}g_{i2}^{log}+\ldots +\beta_{K}g_{iK}^{log}}{K}$$

where $\beta_{k},k=1,2,\ldots ,K$, represents weights and $g_{ik}^{log}=\log\left(g_{ik}+1\right)$ for i=1,2,…,K and k=1,2,…,K represents the log2-transformed cpm-normalized RNAseq expression values. Weights from the differential gene expression in the 80% training sample were applied to expression values in the 20% test sample to generate TAGS. To parallel the age acceleration epigenetic clocks, each TAGS was regressed on chronological age and expression levels of 8 cell-type markers (CD3E, CD3D, CD4, CD8A, CD14, CD19, FCGR3A, and NCAM1)^69^; residuals represent age-acceleration TAGS, with positive values indicating accelerated aging and negative values indicating decelerated aging. TAGS were then correlated with their respective epigenetic clock and tested for associations with aging-related phenotypes (Figure 1, Step 5) in the 20% test sample. Models were adjusted for sex, smoking, ancestry principal components, RNAseq batch, and height (for grip strength and gait speed). Associations were tested using Cox regression for all-cause mortality; linear regression for frailty, BMI, cognitive function, telomere length, grip strength, gait speed, IL-6, and IL-10; Poisson regression for ADLs; and logistic regression for heart problems, diabetes, lung and psychological problems. To assess predictive performance, we fit additional models including both TAGS and their respective DNAm version as joint predictors.

## Supplementary Material

Supplementary Figures

Figures S1. Volcano plots from differential gene expression analysis for each clock

Figures S2. Gene Ontologies (GO) across clocks

Supplementary Tables

Table S1a-e. Differentially expressed genes (gene name, base mean, regression coefficient, p-value) for each clock

Table S2a-e. Reactome pathway enrichment results for each clock

Table S3. Enriched Gene Ontologies (GO) analysis results by clock

Table S4. Test sample characteristics for health phenotypes (n=645)

Table S5. Regression results with both epigenetic and transcriptomic clocks predicting health phenotypes

Supplementary Methods

Supplementary Files

This is a list of supplementary files associated with this preprint. Click to download.

• MssSupplementaryInformation.docx

• MssSITablesS1aeDEGsfdr.01.xlsx

• MssSITablesS2aeGSEAfdr.25.xlsx

• MssSITablesS3GO75clusters.xlsx

## Figures and Tables

**Table 1.** Descriptive Statistics of the DNA Methylation (DNAm) Samples

	Training Sample (n=2,584)			Test Sample (n=645)		
	N	%	Mean (SD)	N	%	Mean (SD)
Race/ethnicity						
White/Caucasian	1,959	75.8		479	74.3	
Black/African American	415	16.1		110	17.1	
Other or Unknown	200	7.7		56	8.7	
Hispanic/Latinx	347	13.4		85	13.2	
Gender						
Female	1,492	57.7		372	57.7	
Male	1,090	42.2		273	42.3	
Smoking Ever (yes)	1,438	55.7		365	56.6	
Chronological Age			70.41 (9.34)			70.12 (9.42)
Age Acceleration DNAm clock						
Horvath			−0.27 (5.43)			−0.24 (5.52)
Hannum			−0.20 (4.76)			−0.28 (4.55)
PhenoAge			0.04 (6.31)			−0.04 (6.23)
GrimAge			0.21 (4.48)			0.31 (4.36)
DunedinPACE			0.00 (0.14)			−0.003 (0.15)

## Data Availability

Public data for the Health and Retirement Study (HRS) can be accessed via the University of Michigan online application (https://hrs.isr.umich.edu/data-products). Sensitive health data from HRS is available under restricted access, and includes data for the Venous Blood Study (VBS) and biomarkers used in the study, including epigenetic clock values. Sensitive health data can be accessed after gaining approval for an application submitted online (https://hrsdata.isr.umich.edu/data-products/sensitive-health). HRS epigenetic and transcriptomic raw data files can be accessed through The National Institute on Aging Genetics of Alzheimer’s Diseases Data Storage Site (NIAGADS) via application and approval (https://hrs.isr.umich.edu/data-products/genetic-data). Further information on the NIAGADS application can be found on its website (https://niagads.scrollhelp.site/support/application-instructions).

## Code Availability

The underlying code for this study is not yet publicly available, but may be made available to qualified researchers on request from the corresponding author.
